# Sonication as a potential tool in the formation of protein-based stable emulsion – Concise review

**DOI:** 10.1016/j.ultsonch.2024.106900

**Published:** 2024-05-20

**Authors:** Harsh B. Jadhav, Pintu Choudhary, Parag Gogate, Seema Ramniwas, Robert Mugabi, Zubair Ahmad, Syed Mohammed Basheeruddin Asdaq, Gulzar Ahmad Nayik

**Affiliations:** a PIHM, Unit UMET, INRAE, 369 Rue Jules Guesde 59650 Villeneuve d’Ascq, France; b Department of Food Engineering and Technology, Institute of Chemical Technology, Matunga, Mumbai, 400019, India; c Department of Food Technology, CBL Government Polytechnic, Bhiwani, Haryana; d Department of Chemical Engineering, Institute of Chemical Technology, Matunga, Mumbai, 400019, India; e University Centre for Research and Development, Chandigarh University, Gharuan, Mohali 140413, Punjab, India; f Department of Food Technology and Nutrition, Makerere University, Kampala, Uganda; g Center of Bee Research and its Products, King Khalid University, P.O. Box 9004, Abha 61413, Saudi Arabia; h Applied College, Mahala Campus, King Khalid University, P.O. Box 9004, Abha 61413, Saudi Arabia; i Department of Pharmacy Practice, College of Pharmacy, AlMaarefa University, Dariyah, 13713, Riyadh, Saudi Arabia; j Department of Food Science & Technology, Govt. Degree College Shopian-192303, J&K, India

**Keywords:** Ultrasound, Emulsion stability, Oil in water, Protein-based emulsion, Oil droplet, Emulsifying characteristics

## Abstract

Emulsion systems are extensively used in the food processing sector and the use of natural emulsifiers like proteins for stabilizing emulsion has been in demand from consumers due to increased awareness about the consumption of healthy food. Numerous methods are available for the preparation of emulsion, but ultrasound got more attention among common methods owing to its economical and environment-friendly characteristics. The physical effects caused by to bursting of the cavity bubble, result in reduced droplet size, thus forming an emulsion with appreciable stability. Ultrasound ameliorates the emulsifying characteristics of natural emulsifiers like protein and improves the storage stability of the emulsion by positively boosting the rheological, emulsifying characteristics, improving zeta potential, and reducing average droplet size. The stability of protein-based emulsion is affected by environmental stresses hence conjugate of protein with polysaccharide showed good emulsifying characteristics. However, the data on the effect of ultrasound parameters on emulsifier properties is lacking and there is a need to develop a sonication device that can carry out large-scale emulsification operation. The review covers the principles and mechanisms of ultrasound-assisted formation of protein-based and protein-based conjugate emulsions. Further, the effect of ultrasound on various characteristics of protein-based emulsion is also explored. This review will provide concise data to the researchers to extend their experiments in the area of ultrasound emulsification which will help in commercializing the technology at the industrial scale.

## Highlights

• Proteins can be used as emulsifiers due to their amphiphilic nature.
• Cavitation events in sonication reduce the droplet size of the emulsion and enhance its stability.
• Ultrasound ameliorates the emulsifying characteristics of natural emulsifiers like protein and improves the storage stability of the emulsion.
• The study on sonication-assisted formation of protein-based stable emulsion is still limited.

## 1 Introduction

The ample array of food products is formulated using emulsion technology by dispersing one phase into another bulk phase. Products like cream, ice cream, mayonnaise, butter, soft drinks, processed milk, flavored milk, desserts, toppings, sauces, etc. are all emulsions of two different phases that are immiscible. The tiny bubbles of one phase are dispersed in the bulk / continuous phase [1]. Since emulsion contains two immiscible liquids, there are high chances of their separation over a period of time. Thermodynamically unstable systems like food emulsion systems can be made stable by using compounds that are active at the interface of two phases and reduce the interfacial tension [2]. The emulsifiers like synthetic anionic/cationic surfactants are generally used to formulate kinetically stable food emulsions. The consumption of food containing synthetic surfactants has a negative impact on human health [3], [4]. The demand from consumers for healthy, natural, and sustainable food has grown in the last decade and hence finding a potential natural substitute for synthetic surfactant has become a need of the hour [1]. Natural biomolecules like proteins can be used as an emulsifiers due to their amphiphilic characteristic, they adsorb at the interface and form a dense layer surrounding the oil droplet thus preventing coalescence [5]. The proteins derived from plant sources have higher molecular weight and limited solubility in the medium, which affects the stability of emulsion stabilized by plant protein [6]. The stresses like ionic strength, pH, and temperature also have a detrimental effect on protein-based emulsion stability.

High-energy methods like ultrasound have attracted the attention of researchers owing to their environment-friendly nature to be a potential approach in the formation of a highly stable protein-based emulsion. The mechanical waves with a frequency of more than 20 kHz are ultrasound, the technique is divided into low-intensity and high-intensity ultrasound. A frequency in the range of 10 – 1000 W/cm^2^ is considered as high-frequency ultrasound and a frequency below 1 W/cm^2^ is low-frequency ultrasound [7]. The physical effects of ultrasounds like acoustic cavitation, and the formation of liquid microjets promote the formation of tiny bubbles in a narrow range of size distribution. The cavitation effect during ultrasound causes formation, growth, and the bursting of a cavitation bubble, the bursting of a cavity bubble builds up the pressure and generates high turbulence which affects the flow properties of the liquid. At the point where the cavity bubble explodes, there is a huge increase in the local temperature and pressure leading to an increase in temperature up to 5000 K and pressure up to 100 MPa, followed by a strong microjet [8]. The propagation of an ultrasonic wave through the medium results in vibration effects and due to the non-homogeneous medium, the vibrational speed of the particle differs, when the ultrasound wave propagates. The variations in the vibrational speed of the particle change pressure and this pressure changes the flow characteristics of the two-phase system. The objective of this review is to highlight the use of ultrasound technology in forming a stable protein-based emulsion. This is a concise review that briefly describes the fundamentals and efficacy of ultrasound in the formation of emulsion, further the consequences of ultrasound on important characteristics of emulsion are also described. The review also covers various protein-based emulsions prepared using ultrasound and the influence of the operating parameters of ultrasound on the emulsion stability. Finally, the future outlook of protein-based emulsion formed using ultrasound is also discussed in brief, which will help the researchers working in this area.

## 2 Fundamentals and efficiency of ultrasound

**Fig. 1:**
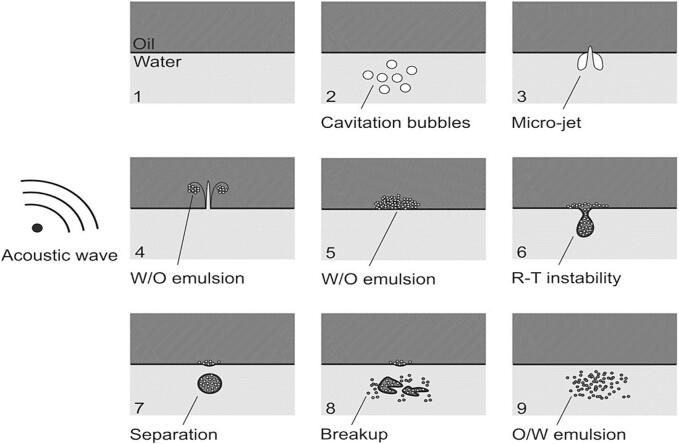
Physical forces responsible for producing stable emulsion (Reproduced with permission from Elsevier, figure adapted from [64].

The significant effect of ultrasound on producing stable emulsion is because of the acoustic cavitation in which formation, growth, and explosion of cavity bubbles result in increasing temperature and pressure inside the system. The changes in the temperature and pressure of the system give rise to physical effects which help in enhancing the stability of the emulsion and chemical effects that can bring about some undesirable changes in the emulsion. Physical forces like shock waves, high pressure, microjet, shear forces, and acoustic streaming are responsible for producing physical outcomes (Figure 1) during the ultrasonication process [9]. Due to the increase in the pressure caused by acoustic cavitation, the larger droplets in the emulsion are broken down into smaller bubbles. The particles in the emulsion, vibrate at different speeds due to the vibration effect caused by the propagation of wave and this divergence in the vibration speed alters the pressure, resulting in changing flow characteristics [10], [11]. The chemical effect of ultrasound causes the formation of free radicals (OH* and H*) by the homolytic breakdown of water molecules in the emulsion. The strong oxidant OH*can oxidize molecules that come in contact with them or they can combine and form H_2_O_2_ [7], [12]. The unsaturated fatty acids and amino acids like cysteine, tryptophan, and histidine can be oxidized due to the formation of free radicals and this oxidation resulted in undesirable changes in the emulsion. Additionally, the increase in the temperature contributed by acoustic waves and by conversion of sound energy into heat energy due to the absorption of sound energy by the system can also cause oxidation of lipids and protein unfolding. This increase in the temperature at various spots within the system can have an impact on the characteristics of the food emulsion. Hence, the physical effects caused due to acoustic process improves the formation of small oil droplets that are mainly responsible for the formation of a stable emulsion.

The process of ultrasound-assisted formation of a stable emulsion depends on the efficiency of the acoustic process. The ultrasound operational parameters ultrasound duty cycle, ultrasound frequency, power, and temperature along with the system parameters including the viscosity of the O/W emulsion can affect the efficiency of the acoustic process. The process of emulsification may not produce stable emulsion due to shorter compression and rarefaction cycles, and much higher frequencies are used for the demulsification process [13]. The frequency in the range of 20 – 40 kHz can be helpful in the formulation of stable emulsion as it promotes the process of emulsification. The increase in the power of the ultrasound process results in the formation of more cavity bubbles which collapse continuously and increase the shear forces which reduce the size of oil droplets in the emulsion and enhance the stability of the emulsion. Li et al. [14] studied the efficiency of the ultrasound emulsification process at different amplitude and sonication times. The authors concluded that the amplitude used in the emulsification process significantly affected the stability of the emulsion as compared with the sonication time. The ultrasound-assisted emulsion also promotes the formation of nanoemulsion by utilizing minimum energy as compared with other non-thermal processes like high-pressure homogenization. The higher duty cycle may also have a negative impact on the emulsification process, a higher duty cycle produces many cavity bubbles that collapse and produce a cushioning effect, thereby negatively affecting the emulsification process. Hence, the optimum combination of frequency (20 – 40 kHz), duty cycle (20 – 50 %), and ultrasound power (250 – 450 W) can be used to formulate an emulsion with higher stability without any undesirable effect on the oxidation of lipids and amino acids present in the emulsion system.

## 3 Consequences of ultrasound on characteristics of emulsion stabilized by protein

### 3.1 Emulsifying stability index

The emulsifying stability index is a measure of the ability of the protein-based emulsifier to form a stable emulsion. Table 1 shows recent studies on the effect of ultrasound on the emulsifying stability index of protein-based emulsions. The emulsion stabilized by α-lactalbumin showed an increase in the emulsifying stability, emulsifying activity, and zeta potential from 50.79 min, 103.23 m^2^g^−1^, 29.4 mV to 63.74 min, 113.38 m^2^g^−1^, 31.8 mV respectively. The increase in the emulsifying stability and emulsifying activity ensures a reduction in the particle size of the emulsion medium [15]. In literature, the stability of the emulsion is also measured using centrifugal stability which refers to the potential of the emulsion system to maintain its stability under adverse conditions. The study reported by Jin et al. [16] showed an increase in centrifugal stability of oil in water emulsion stabilized by 7 s β-conglycinin. The authors reported ultrasound power of 400 w and exposure time of 15 min gave stable emulsion and there was no phase separation in the emulsion stabilized using ultrasound though the particle size of the ultrasound-assisted emulsified sample increased to 238.6 nm from 229.8 nm. The creaming Index of emulsion also gives an idea about the stability of emulsion during storage. The ratio of the height of the upper phase to that of the total height of the emulsion is the creaming index sometimes also referred to as the separation index [17]. The emulsification of oil in water emulsion containing medium chain triglycerides stabilized by soy protein isolate showed a decrease in creaming index. The ultrasound parameters of 40 % duty cycle, 20 kHz, and 18 min gave emulsion with a particle size of 0.5 μm, lower creaming index, and higher stability of ultrasound emulsified emulsion [18]. The stability of the emulsion is also determined by the surface tension, the higher stability of the emulsion is attained with lower surface tension between the two immiscible phases of the emulsion system [19]. Zhu et al. [20] studied the effect of ultrasound on the stability of emulsion (oil in water). The authors reported that ultrasound treatment of 25 kHz for 15 min resulted in increasing the concentration of almond protein at the surface. The ultrasound treatment facilitated the unfolding of protein molecules which increased the adsorption of protein at the surface, enhancing the stability of oil in water emulsion. The recent studies as discussed showed a positive effect of ultrasound on the emulsion stability index. However, still, there is a need to explore more plant proteins as an emulsifier and investigated the effect of different operating parameters on the stability of emulsion (oil in water/water in oil) stabilized by plant protein.

**Table 1:** Recent studies on the effect of ultrasound on the emulsifying stability index.

**Emulsion**	**Emulsion stability**	**Emulsifier**	**Effect of ultrasound**	**References**
Oil in water emulsion	• Emulsifying activity Zeta potential Emulsifying stability Particle size	α-lactalbumin	• Emulsifying activity increased to 113.38 m^2^g^−1^ from 103.23 m^2^g-^1^. Zeta potential increased to 31.8 mV from 29.4 mV Emulsifying stability increased to 63.75 min from 50.79 min. Particle size decreased by 8.94 %.	[15]
Oil in water emulsion	• Centrifugal stability Particle size	β-conglycinin	• Increase in centrifugal stability. Particle size reduced to 229.8 nm and showed no phase separation for 45 days at ambient temperature.	[16]
Oil in water emulsion	• Creaming Index Particle size	Soy protein isolate	• Creaming Index decreased with the application of ultrasound. The particle size was reduced to 0.5 μm.	[18]

### 3.2 Emulsifier

Ultrasound is a high-energy processing operation used to obtain the emulsion with higher efficiency. It is possible that this high-energy process may influence the functional and structural characteristics of protein molecules used as an emulsifier. The ultrasound treatment reduces the droplet size by disintegrating the protein structure, there is an increase in β-sheet and a decrease in the α-helix which exposes the number of hydrophobic groups, thus increasing emulsifying characteristics of protein in protein-stabilized emulsions. In a study, the effect of ultrasound on the emulsifying characteristics of lactoferrin and sodium caseinate was investigated by Furtado et al. [21]. The authors reported that the ultrasound treatment for 2 min resulted in decreasing particle diameter of sodium caseinate and increasing in lactoferrin. Only macroemulsions were obtained in this work since the mean droplet sizes (D3,2) ranged from 1.89 ± 0.06 to 14.68 ± 3.98 mm, immediately after preparation. The changes in the lactoferrin could be due to the formation of non-covalent polymers, reduction in β-sheet, and increased hydrophobicity. Further, the ultrasound effect can form tiny droplets of lactoferrin in the oil in the water system and stabilize the emulsion. Another study showed the effect of ultrasound on the animal and vegetable protein structural, functional, and emulsifying characteristics. The author reported a reduction in the size of all the vegetable and animal proteins except rice protein isolate [22]. The source of proteins and their molecular structure plays a key role in selecting the operating parameters of ultrasound to form a stable emulsion. The textural and emulsion stability is also decided by the oil phase of the emulsion system. The ultrasound effect on the emulsifying characteristics of soy protein isolate containing medium-chain triglyceride and long-chain triglycerides was studied by Taha et al. [18]. The author reported that ultrasound treatment of 18 min for emulsion containing medium chain triglycerides showed a reduced droplet size of 0.5 μm and higher adsorption of protein particles at the interface. The higher hydrophilicity of medium-chain triglycerides resulted in higher adsorption of soy protein isolate at the water–medium chain triglyceride interface.

An increase in ultrasound power also has a negative impact on the emulsifying properties of proteins. The increase in ultrasound power from 150 W to 450 W showed a reduction in the emulsifying potential of proteins. The higher power may result in the formation of insoluble aggregates which had a reduced potential of getting adsorbed at the water–oil interface in emulsion [23]. The ultrasound process reduces the size of oil globules and unfolds protein particles hence it is easier to form emulsion by combining fat with the protein molecule rather than aggregation based on hydrophobic association. The ultrasound-assisted emulsification shows a different effect on the protein structural and functional properties in emulsion than the effect of ultrasound on protein alone. Optimization of ultrasound parameters plays an important role in forming along with the type of protein molecule, its source, and the type of oil phase in forming a stable emulsion. Future studies should undertake the formation of stable emulsion at the pilot scale and check the applicability of results at the industrial scale.

### 3.3 Viscosity of emulsion

The viscosity of the emulsion is an important factor that plays a key role in determining the texture, mouthfeel, and shelf life of food products [24]. The studies reported in the literature showed a decrease in the apparent viscosity with an increase in the ultrasound operating parameters. A recent study by Qayum et al. [15], [15]showed a decrease in the apparent viscosity of oil in water emulsion stabilized using α-lactalbumin. The author measured the apparent viscosity by varying the shear rate from 0.1 to 100 s^−1^. The viscosity of the emulsion decreased to 3.3 Pa s from 3.9 Pa s after exposure of the emulsion to ultrasound for 30 min at 600 W. The decrease in the viscosity of the emulsion subjected to ultrasound could be due to a reduction in the size of the protein particle [25]. The overall viscosity of the emulsion is influenced by the viscosity of interfacial film, the viscosity of the bulk phase, and the size of the droplet. The relation between the viscosity of the bulk phase (ŋ_c_), the radius of the droplet (r), the shear rate (γ), and the interfacial tension (τ) is expressed by Eq. (1);

(1) $$r=\frac{\tau }{\eta cx\gamma }$$

According to Eq. (1) the radius of the droplet is directly dependent on the interfacial tension and indirectly on the shear rate and the viscosity of the bulk phase. The Herschel-Bulkley model given by Eq. (2), gives the value of viscosity (k) and flow characteristics (n) of emulsion

(2) $$\sigma =\sigma 0+kx\gamma^{n}$$

The better fluidity of the emulsion (under external forces) is attained at the larger values of n and lower values of k. The ultrasound-assisted process for the formation of a stable emulsion of soyabean oil resulted in lowering the value of k to 0.0111 Pa s from 0.0164 Pa s and increased the n value to 0.595 from 0.528. The decrease in the size of droplets due to ultrasound treatment increases the flow characteristics of the emulsion and decreases the viscosity of the emulsion [15]. The n and k value of protein-based emulsion subjected to ultrasound also depends on the concentration of oil phase in oil in water emulsion. The increase in the concentration of oil concentration in the oil in water emulsion results in increasing the k value and decreasing the n value, which affects the viscosity and flow behavior of the emulsion. The phenomenon is well explained by Benetti et al. [26] and the authors reported that increasing the concentration of oil to 20 % from 10 % resulted in increasing the k value to 0.39 Pa s from 0.0123 Pa s and a decrease in n value to 0.47 from 0.82 for oil in water emulsion stabilized by microgel of soybean protein isolate. However, some studies show an opposite trend with an increase in the oil concentration of emulsion [27], [28]. The n and k values of the emulsion are highly dependent on the type of emulsion subjected to sonication and the type of protein used as an emulsifier. All these parameters need to be considered while designing a protein-based food emulsion stabilized by ultrasound.

### 3.4 Dimension of the droplet and its electrical characteristics

In a polydisperse emulsion like food emulsion, the size and size distribution of droplets of dispersed phase plays a key role in governing the stability of the emulsion. Excellent stability of the emulsion is achieved with the narrow size distribution of droplets and smaller droplet size. Exposure of emulsion to ultrasound results in the breaking and unfolding of the protein fraction, simultaneously the ultrasound also disrupts the oil droplets present in the emulsion. A dynamic laser size analyzer is used for the determination of Z_avg_ (mean diameter) and the uniformity of the particle size distribution is measured by the polydispersity index (PI) [29]. The Z_avg_ size of droplets present in the emulsion reduces with the increase in ultrasound treatment. The increase in sonication operating parameters, increases the process of acoustic cavitation which helps in reducing the droplet size of the emulsion. The reduction of droplet size to a smaller one enhances the emulsion stability and prevents phase separation. A nanoemulsion stabilized by whey protein showed a decrease in the Z_avg_ size of the particle to 299 nm from 498 nm with an increase in the exposure time of emulsion to ultrasound from 1 min to 4 min. Similar results were observed for the PI, it reduced from 0.75 to 0.42 with an increase in sonication time, thus highlighting the positive effect of ultrasound on the protein-based nanoemulsion [30]. A study on ultrasound-assisted stabilization of human milk fat analog emulsion was reported by Qin et al. [31]. The authors reported a decrease in the Z_avg_ diameter to 187 nm from 202 nm with a variation in the power of ultrasound from 0.06 kW to 0.6 kW. The variation in the ultrasound time from 0 min to 15 min showed a decrease in the PI to 0.17 from 0.23. The higher turbulence generated in the emulsion due to the bursting of the cavity bubble, decreases Z_avg_ diameter and PI, thus making the emulsion more stable. The positive changes in the droplet size of emulsion stabilized by protein under ultrasound conditions can be seen up to an optimum level. Further increases in the ultrasound operating parameters can have a negative impact on the emulsion [23], [32]. A formulation of oil in water emulsion using ultrasound stabilized by whey protein isolate showed a decrease in the Z_avg_ diameter with an increase in ultrasound time from 0 min to 24 min. Exposure of emulsion to ultrasound beyond 24 min resulted in the coalescence of particles, which increased the size of droplets and decreased the stability of the emulsion [23]. The droplet size also depends on the concentration of oil present in the emulsion. The Z_avg_ value increases with an increase in the concentration of oil phase in oil in water emulsion. The increase in the concentration of oil to 16 % from 1 % caused a significant increase in the Z_avg_ value to 8112 nm from 1298 nm [33]. The study on ultrasound-assisted emulsion formation reported in literature stabilized by protein showed varying effects of ultrasound on the size of droplets and their distribution in the emulsion. The operating parameters of ultrasound like power, sonication time, type of ultrasound used i.e., ultrasound probe/ultrasound bath, and the concentration of oil in the emulsion are the deciding factors that decide the size of droplets and their distribution, which directly affect the stability of the emulsion.

The adsorption of the emulsifier molecule on the surface of the droplet gives it an electrical charge and the charge on the droplet has a significant influence on the stability of emulsion. The charge on the emulsion droplet has an impact on its association with the other components present in food emulsion and also decides the ability of the droplet to cohere with biological surfaces [34]. The electrical properties of the droplet in emulsion are investigated by estimation of zeta-potential value. The value of zeta potential higher than 30 mV indicates higher stability of the emulsion. The effect of ultrasound on the zeta potential is different for different types of emulsion formulations. For example, the ultrasound emulsification of soyabean oil emulsion using α-lactalbumin [15], sunflower oil emulsion using lactoglobulin [35], human milk fat analogs stabilized by whey protein isolate [31] showed increased in the value of zeta potential after ultrasound treatment. However, for some emulsions, there was no change in the zeta potential [36] and for some emulsions, there was a decrease in the zeta potential of emulsion after ultrasonication.

## 4 Ultrasound-assisted protein-based emulsions

### 4.1 Emulsion stabilized by proteins

The protein molecule is a good emulsifier owing to its amphiphilic characteristics because of the presence of hydrophobic and hydrophilic amino acid residue. The presence of both the hydrophobic/philic amino acid in the protein molecule helps in reducing the interfacial tension between two immiscible fluids, thus enhancing the stability of protein-based food emulsion during processing and storage [37]. Regardless of the amphiphilic characteristics of the protein, there are some disadvantages associated with protein as an emulsifier. Some proteins have limited solubility, high molecular weight, and hydrophobicity, also some proteins have weak electrostatic repulsion which negatively affects the emulsifying characteristics of the protein. These limitations associated with proteins can be overcome by using high-energy, non-thermal technology like ultrasound, which possibly forms emulsions with higher stability and good characteristics. The exposure of emulsion to ultrasound reduces droplet size and forms droplets with narrow size distribution. The ultrasound enhances the solubility of proteins and increases their adsorption ability at the oil–water interface in the emulsion. The cavitation effect in the ultrasound exposes a more hydrophobic group of proteins by altering the tertiary structure of the protein. The availability of a more hydrophobic group at the interface enhances the flexibility of protein molecules, thereby forming a stable film at the interface, which provides more strength to the emulsion. The application of ultrasound in the formation of protein-based stable emulsion is shown in Table 2. Shanmugam et al. [24] reported the application of ultrasound with 20 kHz frequency, 178 W power, and 1 – 8 min exposure time in the formation of flaxseed oil emulsion. The authors reported a reduction in the size of droplets with an increase in the exposure time of emulsion to ultrasound. The smallest size of 0.40 μm was achieved at an exposure time of 8 min giving higher stability to the emulsion as compared with emulsion with an exposure time of 1 or 2 min. Exposure of emulsion to ultrasound for a longer duration allows it to come in contact with the high shear forces produced due to the cavitation effect which reduces the size of droplets and forms a stable emulsion. The increase in the exposure time of emulsion to ultrasound allowed protein to boost their solubility, so that more protein molecules got a chance to adsorb at the oil–water interface, thereby enhancing the stability of the emulsion [18]. A grape seed oil emulsion was stabilized using different combinations of casein and whey at different energies. The authors used different combinations of whey/casein (80/20, 60/40, 50/50, and 40/60) as an emulsifier for stabilizing grape seed oil using ultrasound. The increase in the energy density of ultrasound during emulsification resulted in the formation of smaller droplet sizes for all the combinations of whey/casein. However, the minimum energy required to form smaller size droplets was different for all the combinations of whey/casein [38]. Higher intensity of ultrasound used for emulsification can also result in changes in the thiol group due to the oxidation of protein resulting in the formation of disulfide bonds. This change facilitates the formation of a strong interfacial layer of protein, which further produces a stable emulsion [39].

**Table 2:** Recent studies on protein-based emulsion stabilized using ultrasound.

**Dispersed phase**	**Emulsifier**	**Ultrasound operating parameters**	**Conclusion**	**References**
Turmeric oil	Sodium dodecyl sulphate	Ultrasound frequency (UF) – 20 kHz, Ultrasound power (UP) – 135 W, Ultrasound exposure time (UT) – 5 min	Ultrasound treatment to 300 mL capacity mixture resulted in formation of stable emulsion with mean droplet diameter of 232.6 nm.	[48]
Medium and long chain triglycerides	Soyabean protein isolate	UF – 20 kHz, UP – 50 – 55 W/cm^2^, UT – 2 – 18 min	Ultrasound reduced the droplet size of emulsion to 0.5 μm. The ultrasound energy density of 1620 J/mL for 18 min formed the stable emulsion which showed stability at room temperature.	[18]
Clove essential oil	Hazelnut meal protein	UF – 20 kHz UP – 750 W UT – 0 – 6 min	Ultrasound resulted in formation of stable emulsion with Z_avg_, PI and zeta potential values as 160.45 nm, 0.277 and −9.975 mV respectively.	[49]
Corn oil	Almond protein	UF – 25 kHz UT – 15 min UP − 200 – 600 W	Ultrasound emulsification resulted in reducing the droplet size less than 200 nm, increased adsorption of protein molecule at the interface resulting in increased stability of emulsion.	[20]
Canola oil	Pea protein	UT – 5 min	Droplet size (D_4,3_) reduced to 90 nm after ultrasonication of emulsion.	[50]
Rapeseed oil	• Soy protein isolate Whey protein isolate Gelatin	UF – 20 kHz UT – 6 min UP – 540 J/mL	• The droplet size was reduced to 1.24 μm (D_4,3_) and 0.69 μm (D_3,2_). The droplet size was reduced to 1.15 μm (D_4,3_) and 0.61 μm (D_3,2_). The droplet size was reduced to 1.06 μm (D_4,3_) and 0.51 μm (D_3,2_).	[40]
Sunflower oil	Soybean protein	UP – 500 W UT – 9 min	The ultrasound emulsification reduced the particle size with the Z_avg_ value of 291.6 nm.	[51]
Sunflower oil	Pea protein	UF – 20 kHz UP – 300 W UT – 5 min	The sonicated sample containing 0.5 % emulsifier showed creaming index of 3.87 %, particle size of 2.85 μm, size of fat droplet 0.51 μm.	[52]
Grapeseed oil	Milk protein	UF – 20 kHz UP − 300 W UT – 1 – 10 min	The sonication process gave droplet size of 0.5 – 3.2	[53]
Soyabean oil	Rice bran protein	UF – 20 kHz UP – 65–195 W UT – 20 min	Z_avg_ value of 1.23 – 2.38 μm was obtained after the ultrasound exposure of emulsion for 20 min.	[54]
Sunflower oil	Whey protein isolate	UF – 20 kHz UP – 600 W UT – 9 min	The mean droplet diameter (MD) decreased with increase in the sonication time. The MD decreased from 216.5 nm to 131.5 nm with increase in sonication time from 3 min to 33 min.	[55,56]
Rapeseed oil	Whey protein isolate	UF – 20 kHz UP – 130 W UT – 2–10 min Duty cycle – 10 %	The emulsion with the particle size of 0.43 μm is obtained after the sonication treatment of emulsion.	[57]
Soyabean oil	Pea protein isolate	UF – 20 kHz UP – 525 W UT – 4 min	Decrease in the protein size and increase in the emulsifying activity index was observed for ultrasound treatment with power/time combinations of 412.5 W/581.82 s, 487.5 W/492.31 s, 562.5 W/426.66 s.	[58]
Soyabean oil	Antarctic krill protein	UP – 6690 W UT – 2 min	The zeta potential of emulsion after ultrasound was in the range of + 6 − +17 mV, with the diameter of particle around 5 μm	[59]
Chia seed oil	Pectin and whey protein concentrate	UF – 20 kHz UP – 128 W UT – 2 min	The minimum particle size of 1.02 μm is obtained after sonication and using protein-polysaccharide conjugate as an emulsifier. There was no phase separation observed for the emulsion.	[60]
Soyabean oil	Pectin and whey protein concentrate	UF – 20 kHz UP – 120 W UT – 1 min	The droplet size of 1.88 μm (D_3,2_) was obtained after sonication.	[44]
Palm oil and resveratrol	Corn starch hydrolysates and sodium caseinate	UF – 20 kHz UP – 150 W UT – 7 min	The D_4,3_ and D_3,2_ droplet size of final emulsion was 1.49 μm and 0.46 μm respectively.	[61]
Sunflower seed oil	Potato starch and Gelatin	UF – 25 kHz UP – 150 W UT – 5–15 min (Ultrasound bath)	The D_4,3_ droplet size of final emulsion was in the range of 2.3 – 2.7 μm. The obtained emulsion showed no phase separation for 7 days.	[62]
Coconut cream	Maize starch and coconut milk containing 3.9 % protein	UF – 20 kHz UP – 50–55 W/cm^2^ UT – 13 min	The initial droplet size of 60–300 μm was reduced to smaller particle size falling in the range of 0.01 – 0.7 μm after ultrasound treatment	[63]

**Fig. 2:**
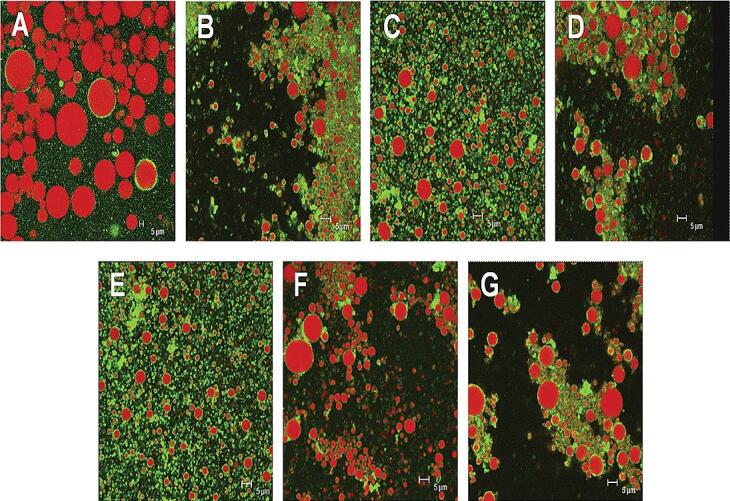
Microscopy images of emulsion stabilized by protein with the application of ultrasound, (A) control sample, (B) 150W/12min, (C) 300W/12min, (D) 450W/12min, (E) 150 W/24min, (F) 300W/24min, (G) 450W/24min (Reproduced with permission from Elsevier, figure adapted [23]

The positive effect of ultrasound as a high-energy emulsification process to form stable protein-based emulsions is achieved up to an optimum level of operating parameters. Increasing ultrasound intensity beyond the optimum range results in adverse effects on the emulsion, reducing the stability of the emulsion. A study reported by Sui et al. [23] showed an increase in the droplet size of emulsion by 79.25 % with increasing ultrasound power to 450 W. The increase in the ultrasound power destroys the secondary structure of the protein molecule, which results in the formation of insoluble aggregates of the protein molecule hence the amount of protein molecule adsorbed at the interface decreases, which negatively affects the stability of the emulsion. As seen in Figure 2, the control sample ‘A’ shows less stability, with an increase in the power intensity from 150 to 450 W (12 min) the size of the droplet increases, as seen from samples B, C, D. Similarly, the stability of emulsion is affected with increase in power from 150 to 450 W (24 min) as seen from sample E, F, G. The protein molecules from animal and plant sources respond differently to the ultrasound intensity, future research in formation of stable emulsion under ultrasound can help in achieving the goal of sustainability.

### 4.2 Protein-based secondary emulsions

**Fig. 3:**
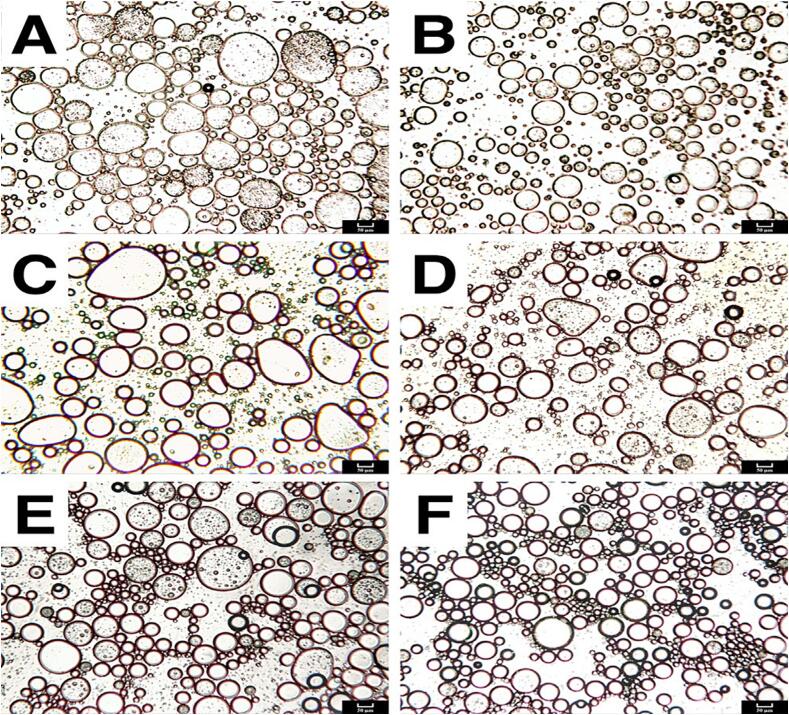
Optical microscopy images of (A) emulsion stabilized using soy protein isolate, (B) ultrasound assited emulsification using soyprotein isolate. (C) emulsion stabilized using citrus pectin, (D) ultrasound assited emulsification using citrus pectin, (E) emulsion stabilized using soy protein isolate- citurs pectin conjugate, (F) emulsion stabilized using soy protein isolate- citurs pectin conjugate under presence of ultrasound. (Reproduce with permission from Elseveir, Adapted from [42].

The proteins are used as emulsifiers in the formulation of emulsions with smaller droplet sizes and higher stability, but these protein-based emulsions are sensitive to ionic strengths, temperatures, and changes in pH. However, the emulsions formed using polysaccharides as emulsifiers are stable against these environmental stresses. Hence the formulation of a protein-based secondary emulsion using polysaccharide (PO) and protein (PR) as an emulsifier could form emulsions having smaller droplet sizes and resistant to environmental stresses [40], [41]. The synergistic effect of PO and PR conjugates formed with the covalent/non-covalent interaction forms an emulsion with higher stability as explained in a detailed review by Rodriguez Patino & Pilosof, (2011). Table 2 shows some recent studies on emulsion stabilized by PO-PR with the application of ultrasound. A study by Ma et al. [43] investigated ultrasound-assisted formation of emulsion stabilized by pectin (extracted from citrus) and soy protein isolate. The ultrasound power of 630 W for 10 min resulted in enhancing the emulsifying activity index by 44.1 % and the emulsifying stability index by 24.68 %. The effect of ultrasound and the synergistic effect of PO-PR can be well understood in Figure 3. Figure 3A shows droplets with irregular and larger sizes as stabilized only by using soy protein isolates. Figure 3B showed ultrasound-assisted formation of emulsion using soy protein isolate, in which droplet size has reduced considerably giving a relatively stable emulsion. The emulsion formed without ultrasound and using PO-PR conjugate formed small droplets with thick walls, but still, large droplets could be spotted in Figure 3E. With the introduction of ultrasound and PO-PR conjugate as an emulsifier, the emulsion showed the formation of smaller droplets having narrow size distribution (Figure 3F) forming a more stable emulsion. The sonication process promotes the graft reaction between the polysaccharide and protein as reported by Li et al. [44]. The authors concluded that the sonication process significantly promoted the graft reaction between pea protein isolate and glucomannan, which enhanced the availability of free amino acids from pea protein isolate. The sonication process improved the solubility and emulsifying characteristics of the preprotein-glucomannan conjugate, increased in β-helix, reduced in α-helix, and improved hydrophobicity of conjugate as compared with the conventional heating process. The ultrasound process improves the electrostatic interaction between the PO-PR conjugates, thereby improving the emulsifying characteristics of the emulsion for a long duration. The ultrasound parameters of 20 kHz frequency and 120 W power for the 60 s resulted in improved electrostatic interaction between pectin and whey protein isolate, thus improving the emulsifying characteristics of emulsion by forming small droplets with narrow size distribution. The formed emulsion was stable for 7 days without any phase separation at ambient temperature [45]. Ultrasound treatment to PO-PR conjugate emulsion reduces the viscosity of the conjugate which facilitates quick adsorption of this conjugate at the interface which reduces interfacial tension between two immiscible phases and forms a final emulsion with higher stability. In conclusion, sonicating conjugate reduces the size of protein molecule thereby exposing more hydrophobic groups of protein making it available at the interface and the reactions like glycosylation, and acetalization in polysaccharide improves solubility of polysaccharide which upgrades the emulsifying potential of PO-PR conjugate forming a more stable food emulsion.

Synthetic surfactants are amphiphilic molecules with smaller molecular weights that are used to stabilize emulsions in the food processing sector. Due to the presence of both the hydrophilic/phobic end and small molecular weight, surfactants can adsorb themselves easily and quickly at the oil–water interface, preventing coalescence and forming a highly stable food emulsion. The surfactants that are mostly used in the preparation of food emulsions include lecithin, polyglycerol polyricinoleate, Tween 20/40/80, and span80. The advantages of surfactant can be utilized by adding it to protein and forming a conjugate which could help in forming a highly stable emulsion. The combination of surfactant and protein could be a better option for the formulation of a highly stable emulsion. However, there are no studies reported in the literature on protein-surfactant combination as an emulsifier in stabilizing food emulsions. The studies reported in the literature show the individual effects of surfactants and proteins as emulsifiers on the formulation of emulsion with the application of ultrasound [29], [31], [46]. Future research in this approach would help in understanding the effect of ultrasound parameters like power, exposure time, duty cycle, and frequency on the emulsion stabilized by the combination of protein and surfactant. Further, the consequences of different combinations of protein and substrate can be studied in emulsifying oil in water food emulsion.

## 5 Consequences of ultrasound parameters on emulsion

Ultrasound has gained popularity owing to its eco-friendly and energy-efficient characteristics. In the food processing sector, ultrasound has been found useful in several applications which start from pre-treatment to raw material to preservation of final processed food product [11]. Ultrasound-based emulsion formation has also become a point of attraction for food researchers working in the area of fruit juices, products like mayonnaise, dairy products, meat products, encapsulation of aroma/flavor compounds, etc. [10], [47]. The physical and chemical effects generated during ultrasonication of emulsion positively modify the emulsification characteristics of emulsion and enhance their stability /shelf-life. The physical effects of ultrasound generate high turbulent energy which reduces the droplet size of the emulsion and produces droplets with a narrow size distribution, these changes in droplet size increase the zeta potential and flow index and decrease the apparent viscosity of the emulsion, thus forming a stable emulsion. This is achieved with optimum ultrasound operating parameters like frequency, power, and sonication time. An increase in these operating parameters beyond an optimum point, affects the emulsion negatively leading to reduced emulsion stability. The critical review of the literature revealed that the most critical parameters affecting the ultrasound emulsification process are power and time. Most of the emulsification process assisted by ultrasound is carried out at a fixed frequency of 20 kHz, hence frequency is not considered a critical parameter. Sui et al. [23] have studied the effect of increasing ultrasound power and sonication time beyond the optimum point on emulsion stability. The author reported increasing ultrasound power from 150 W to 450 W, initially increased the emulsifying stability index and emulsifying activity index to 221.03 min and 149.23 g/m^2^ which reduced to 52.03 min and 93.98 g/m^2^ respectively when power was increased beyond 300 W. Similarly, increasing sonication time from 0 min to 24 min, initially reduced the droplet size from 3.815 to 3.369 μm till 12 min, increasing exposure time beyond 12 min significantly increased the droplet size due to the coalescence of droplets, which negatively affected the stability of the emulsion. The increase in the power of ultrasound enhances the intensity of ultrasound forces, which takes the emulsion droplets to the antinodes and nodes of the cavitation field. The gathering of droplets at this proximity results in the coalescence of these droplets forming a larger droplet, thereby reducing the stability of the emulsion [32]. Similarly, a recent study by Rajasekaran et al. [48] reported the effect of ultrasound amplitude and sonication time on the fish oil n water emulsion. The author reported increased solubility of shrimp myofibrillar protein with an increase in the amplitude from 40 % to 60 % showing decreased solubility of shrimp myofibrillar protein, caused due to adverse due to ultrasound. The intense cavitation effect and shockwave may result in the denaturation of protein structure and expose a hydrophobic group of shrimp myofibrillar protein which caused aggregation of protein molecules by hydrophobic interaction of protein molecule, which decreased their solubility and also negatively altered the interaction of shrimp myofibrillar protein with the oil droplets in emulsion, which also affected the emulsion stability index and emulsion activity index. The optimization of ultrasound parameters is a critical factor in applying ultrasound to form stable protein-based emulsions. The studies reported in the literature have optimized parameters for the laboratory-scale emulsification of emulsion using protein under the presence of ultrasound. However, data are scarce on the optimization of ultrasound parameters for large-scale emulsification at an industrial scale. Future studies in this area should undertake the formation of protein-based emulsion under ultrasound at pilot scale/large scale, which will help in the production of protein-based emulsion at the commercial level.

## 6 Concluding remark and future outlook

Ultrasonication technology has come into the limelight in the 21st century owing to its environment-friendly features and it is used for various applications in the food processing sector. The consumers across globe are in demand of a potential replacement for synthetic emulsifiers used in food products, protein molecules being amphiphilic and natural in origin have emerged as a healthy replacement for synthetic emulsifiers. Much research reported in the literature has used ultrasound as a tool for the formation of protein-based emulsions. The obtained results from these studies have shown that ultrasound is a good emulsification tool to produce a stable food emulsion. However, the stability of food emulsion largely depends on the type of emulsifier used and the operating parameters of ultrasound. The research on the protein-stabilized emulsion using ultrasound is in its infant stage and much future research in this area needs to be carried out for its commercialization. More research should be carried out to investigate the effect of the combination of fibular and globular protein on the stability of food emulsion. The better stability of emulsion could be achieved by the synergistic effect of PO-PR conjugate which is stable to the environmental stresses as compared with the protein alone. The studies published in the literature have only focused on the effect of ultrasound on the emulsion stability index, droplet size, zeta potential, and emulsion activity index but there is no data available on the effect of ultrasound on the characteristics of emulsifier. The dispersed phase of food emulsions reported in the literature is vegetable oil, but the literature has no information on the solid fat used in the meat processing industry, hence the upcoming research in this domain should also focus on the use of protein from meat as a stabilizing agent for animal fat-based emulsion, this will help in increasing the horizon of this technology in meat processing industries. Lastly, the proper equipment solely dedicated to ultrasound-based emulsification using natural emulsifiers should be designed by a proper collaboration of research institutes with the industries, which will also help in commercializing the process and help in the formation of stable food emulsions at the industrial scale.

## 7 Author agreement statement

We all the coauthors declare that this manuscript is original, has not been published before and is not currently being considered for publication elsewhere. We confirm that the manuscript has been read and approved by all named authors and that there are no other persons who satisfied the criteria for authorship but are not listed. We further confirm that the order of authors listed in the manuscript has been approved by all of us. We understand that the Corresponding Author is the sole contact for the Editorial process. He/she is responsible for communicating with the other authors about progress, submissions of revisions and final approval of proofs.

## CRediT authorship contribution statement

**Harsh B. Jadhav:** Writing – review & editing, Writing – original draft, Supervision, Methodology, Investigation, Formal analysis, Conceptualization. **Pintu Choudhary:** Writing – review & editing, Writing – original draft, Supervision, Software, Resources, Investigation, Funding acquisition, Formal analysis. **Parag Gogate:** Writing – review & editing, Writing – original draft, Validation, Software, Resources, Conceptualization. **Seema Ramniwas:** Writing – review & editing, Software, Resources, Methodology, Data curation. **Robert Mugabi:** Writing – review & editing, Writing – original draft, Visualization, Validation, Software, Methodology, Funding acquisition. **Zubair Ahmad:** Conceptualization, Data curation, Funding acquisition, Project administration, Supervision, Writing – original draft. **Syed Mohammed Basheeruddin Asdaq:** Data curation, Formal analysis, Funding acquisition, Methodology, Resources, Validation, Writing – review & editing. **Gulzar Ahmad Nayik:** Writing – review & editing, Writing – original draft, Supervision, Software, Resources, Data curation, Conceptualization.

## Declaration of competing interest

The authors declare that they have no known competing financial interests or personal relationships that could have appeared to influence the work reported in this paper.

## Data Availability

Data will be made available on request.
